# Complete Genome Sequences of Mycobacterium smegmatis Phages NihilNomen and Carlyle, Isolated in Las Vegas, Nevada

**DOI:** 10.1128/MRA.00677-19

**Published:** 2019-09-19

**Authors:** Alicia Salisbury, Ryan Doss, Astha Mehta, Khadija Bhatti, Ciera Dapra, Audrey Huntsinger, Stephanie Rodriguez, Scott Yacek, Rylee Sandberg, Alexis Gildore, Jacinda Knudtson, Frances Tibayan, Tiannah Ohta, Neha Zafar, Guadalupe Mercado, Alan Le, Natalie Mekhaeel, Justin Willer, Edith Rodrich-Zuniga, Merissa McFarland, Kurt Regner, Christy Strong, Philippos K. Tsourkas

**Affiliations:** a School of Life Sciences, University of Nevada Las Vegas, Las Vegas, Nevada, USA; DOE Joint Genome Institute

## Abstract

We present the complete genomes of the Mycobacterium smegmatis phages Carlyle and NihilNomen, isolated from soil in Las Vegas, Nevada. The phages were isolated and annotated by undergraduate students enrolled in the Phage Discovery course offered by the School of Life Sciences at the University of Nevada Las Vegas.

## ANNOUNCEMENT

Phages that infect Mycobacterium smegmatis account for the largest number of sequenced phage genomes, numbering approximately 1,800 (1). This is largely due to the versatility of this host and its consequent popularity with the Howard Hughes Medical Institute’s (HHMI) Science Education Alliance-Phage Hunters Advancing Genomic and Evolutionary Science (SEA-PHAGES) program (2). Recently, three M. smegmatis phages isolated by students in the SEA-PHAGES program were used to treat a potentially lethal infection of antibiotic-resistant Mycobacterium abscessus in a cystic fibrosis patient (3). Here, we present the complete genomes of two M. smegmatis phages isolated by students enrolled in the Phage Discovery course (BIOL 207X and BIOL 209X) at the University of Nevada Las Vegas (UNLV).

Both phages were isolated from soil obtained from the community gardens on the UNLV campus by students in the course BIOL 207X. A direct isolation method was used. Environmental samples were incubated with enrichment broth and shaken (250 rpm, 2 h) at room temperature, followed by centrifugation and filter sterilization (0.22 μm) of the supernatant per HHMI’s SEA-PHAGES Phage Discovery Guide (https://seaphages.org/faculty/information/#phagediscovery). The phages were then purified and amplified in M. smegmatis mc^2^155. The DNA was extracted using the manufacturer’s protocol, provided in the phage DNA isolation kit (catalog number 46800; Norgen Biotek). Phage genomes were sequenced at the University of Pittsburgh. Sequencing libraries were prepared from genomic DNA by using an NEB Ultra II kit, producing 150-bp single-end reads. The libraries were sequenced with an Illumina MiSeq instrument, yielding single-end reads sufficient to provide at least 150-fold coverage for each genome. The reads were quality trimmed and assembled *de novo* by using Newbler version 2.9 with default settings, in each case yielding a single contig, which was checked for completeness, accuracy, and phage genomic termini by using Consed version 29, as described by Russell (4).

The assembly results (coverage depth, genome length, GC content, number of genes) and the phages’ GenBank and SRA accession numbers are shown in Table 1. The phages were assigned to clusters based on genomic sequence similarity using the phagesdb.org database and Phamerator software with default settings (1, 5). Despite their similar geographic provenance, the phages are not closely related. Carlyle is cluster A1 and NihilNomen is cluster J based on the average nucleotide sequence identity. A ClustalW multiple alignment using default settings showed that the phages have roughly 45% average nucleotide sequence identity (ANI) between them, which is the same as that of two randomly generated DNA sequences. Both phages are predicted to use the “cohesive ends with 3′ overhangs” DNA packaging strategy (6).

**TABLE 1:** Phage GenBank and SRA accession numbers and genome assembly results

Phage name	GenBank accession no.	SRA accession no.	Avg coverage	Cluster	Genome length (bp)	GC content (%)	No. of genes
Carlyle	MK967401	SRX5960826	1,929	A1	51,220	63.6	91
NihilNomen	MK967402	SRX5960825	897	J	11,0439	60.8	241

The assembled genomes were annotated using DNA Master with default settings, as described by Pope and Jacobs-Sera (7), by students in the course BIOL 209X at UNLV. We identified 91 genes in Carlyle and 241 in NihilNomen, of which 1 is a tRNA (Gly-tcc). The protein functions were assigned using Protein BLAST (blast.ncbi.nlm.nih.gov/Blast.cgi?PAGE=Proteins), Batch Web CD-Search (ncbi.nlm.nih.gov/Structure/bwrpsb/bwrpsb.cgi), and HHpred (toolkit.tuebingen.mpg.de/tools/hhpred) using default settings. Using a cutoff E value of 1E-7, we assigned putative functions to 46 genes in Carlyle (50.5%) and 65 genes in NihilNomen (27%). A small and large terminase, a portal protein, a capsid maturation protease, a major capsid protein, a major tail protein, two tail assembly chaperones, a tail tape measure protein, lysin A and lysin B, and integrase were identified in both phages. The tail assembly chaperones have a predicted translational frameshift located in the 3′ region of the upstream tail assembly protein. A holin, excise, and Cro were identified in NihilNomen but not in Carlyle. Of interest is the identification of a 690-bp-long third terminase (gp2) in NihilNomen, an unusual feature present in some cluster J M. smegmatis phages, which is currently being investigated in our laboratory.

### Data availability.

GenBank and SRA accession numbers are listed in Table 1.
